# Cats in Positive Energy Balance Have Lower Rates of Adipose Gain When Fed Diets Containing 188 versus 121 ppm L-Carnitine

**DOI:** 10.1155/2016/2649093

**Published:** 2016-08-29

**Authors:** M. A. Gooding, D. L. Minikhiem, A. K. Shoveller

**Affiliations:** ^1^The Iams Company, Mars PetCare, 6571 State Route 503 North, Lewisburg, OH 45338, USA; ^2^Centre for Nutrition Modelling, Department of Animal Biosciences, University of Guelph, 50 Stone Road East, Guelph, ON, Canada N1G 2W1

## Abstract

L-carnitine (LC) is included in select adult feline diets for weight management. This study investigated whether feeding adult cats with diets containing either 188 ppm of LC (LC188) or 121 ppm of LC (LC121) and feeding them 120% of maintenance energy requirement (MER) resulted in differences in total energy expenditure (EE), metabolic fuel selection, BW, body composition, and behavior. Cats (*n* = 20, 4 ± 1.2 yrs) were stratified for BCS and randomly assigned to one of two dietary treatments and fed for 16 weeks. BW was measured weekly, and indirect calorimetry, body composition, physical activity, play motivation, and cognition were measured at baseline and throughout the study. A mixed, repeated measures, ANCOVA model was used. Cats in both treatments gained BW (*P* < 0.05) throughout the study, with no differences between treatments at any time point (*P* > 0.05). There were no differences in body composition between groups at baseline; however, body fat (g) and body fat : lean mass ratio were greater in cats fed LC121 in contrast to cats fed LC188 (*P* < 0.05) on week 16. No other outcomes differed between treatments (*P* > 0.05). Supplying dietary LC at a dose of at least 188 ppm may be beneficial for the health and well-being of cats fed above MER.

## 1. Introduction

While there is no dietary requirement for L-carnitine (LC) in cats, as it is synthesized endogenously, LC is considered a conditionally essential nutrient, as deficiencies can occur during certain disease states, during aging, and during weight loss/gain, as LC facilitates fatty acid metabolism [1] and energy metabolism [2]. Further, LC is believed to enhance cognition in humans [3] and animal models, such as rats demonstrating cognitive impairments [4]. L-carnitine is a cofactor that facilitates the transport of long chain fatty acids (LCFA) across the inner mitochondrial membrane for subsequent *β*-oxidation. Furthermore, LC also acts as a cofactor in the transport of acetyl-CoA out of the mitochondria. Increased concentrations of mitochondrial acetyl-CoA can inhibit further *β*-oxidation. Together these actions regulate the intramitochondrial acetyl-CoA concentrations and release free CoA and acetyl-carnitine that favor the oxidation of pyruvate. Due to the mechanism of action by which LC exerts its effects on fatty acid metabolism, LC may also provide a mechanism for removal of excessive fatty acids that are released during weight loss [2]. This is critically important in cats because lipids released during weight loss are commonly deposited in the liver if they are not oxidized and result in hepatic lipidosis [5]. Weight gain and related metabolic indices in domestic cats are related to diminished physical activity, mainly in the light hours [6]. Similarly, obese cats have lower EE than lean cats [7], and we recently found that overweight and obese cats have reduced activity counts in contrast to lean cats [8]. Recently, dietary LC (100 ppm) fed to cats has been shown to increase EE and lipid oxidation in contrast to cats fed control (30 ppm) during controlled weight loss [9] and for overweight cats fed to weight maintenance [7].

We have previously demonstrated that dietary LC supplementation can positively impact motivation to play in overweight, but not lean, cats [7]. We hypothesized that LC, partly through actions on energy metabolism and metabolic function, influences central energy sensing that is thought to be part of the underlying control system of predation and play in the cat. In the cat, it is hypothesized that appetitive components of play are related to energy metabolism without an influence on consummatory features once the toy was acquired [10]. Indeed, previous observations in rodents found that LC protected against chronic stress effects and was correlated with a reduction in dopamine to support normal appetitive behavior during food reward trials [11]. In aged dogs, LC has been shown to impact behavior and brain function specifically related to an observed decline in brain function with aging [12] but not after short-term supplementation [13]. Lastly, LC administration has also been demonstrated to increase voluntary physical activity levels when fed to aged rats [14]. It is unclear as to what is the most efficacious level of LC, the population to produce a positive behavioral response, and the appropriate feeding regime to produce the greatest effect.

Previous data suggested that the addition of LC to a maintenance diet had a beneficial effect on supporting healthy metabolism and behavior in overweight, but not lean, cats [7]. Our previous study investigated the effects of LC when cats were fed to maintain body weight, but we do not have any data on the effects of dietary LC when cats are fed above maintenance energy requirement (MER), as often occurs in home environments. In addition, the efficacy of dietary LC has generally been investigated during weight loss [9]. Therefore, the primary objective of the present study was to further investigate the effects of dietary LC on energy and macronutrient metabolism and motivation to play. Results from our previous study were used to power the present study. Our secondary objective was to understand the effects of LC on weight control and body composition. We hypothesized that cats fed a diet containing the higher of two LC concentrations tested would have greater energy expenditure, lower adipose gain, and improved behavioral markers as defined by improved physical activity, play motivation, and cognition when fed above maintenance energy requirements. We chose to investigate two levels of LC, 120 and 200 ppm, as these are two dietary concentrations commonly found in commercial diets. Rather than providing data versus no additional dietary LC, we wanted to understand whether two different supplemental levels of LC provided added benefits.

## 2. Experimental Methods

All procedures were reviewed and approved by the Institutional Animal Care and Use Committee at the Iams Company, Procter & Gamble Pet Care.

### 2.1. Experimental Design

Twenty neutered/spayed cats (*N* = 20; M = 11 and F = 9) of similar age (4 ± 1.2 years) were randomly assigned to treatment groups balanced for body weight (Group A: BW = 4.48 kg ± 1.15; Group B: BW = 4.50 kg ± 0.99) and maintenance energy intake (Group A: caloric intake = 44.85 kcal ME/kg BW ± 6.96; Group B: caloric intake = 45.85 kcal ME/kg BW ± 0.99). Cats were fed a control diet (CON), with no supplemental LC, for 4 weeks prior to the initiation of the study to allow for washout and baseline measures. Following baseline assessments, groups were stratified based on BW and assigned to receive either the control diet with 121 ppm LC (LC121) or the control diet with 188 ppm LC (LC188) in a parallel study design for a total of 16 weeks.

### 2.2. Diets

Dry diets were based on Iams ProActive Health Original with Chicken without any L-carnitine tartrate for the baseline period (~30 ppm of endogenous LC), with current supplemental L-carnitine tartrate (LC121), and with added L-carnitine tartrate (LC188) (Carniking*™*, Lonza Group Ltd.) (Table 1), respectively. Diets were identical formulations and utilized identical batches of ingredients; therefore, minimal differences in macronutrient (protein, fat, ash, and fiber) and micronutrient concentrations are assumed. Final products tested included a total of 121 ppm and 188 ppm LC as analyzed. Each cat was fed to mimic consumer relevant feeding practices while controlling the amount of caloric surplus; thus, cats were offered 120% of their total daily energy requirement (kcal ME/kg BW) and adjusted weekly based on BW. Individual energy requirements were established based on historical records of the individual dietary energy required to maintain body weight and BCS because the management of this colony of cats has been to maintain body condition between BCS of 2.5 and 4.0 and incur little loss or gain of body weight (no more than ±5%). Diets were presented in dry, kibble form and cats were fed once daily individually at 7:00 a.m. and given 60 minutes to eat during food offerings. All remaining food was collected and weighed to account for total (grams) food refusal. On cognition testing days feeding programs were altered for individual cats undergoing testing as food was used as a reward. Cats were fed 25% of their total daily allotment at 7:00 a.m. and the residual 75% of food was offered during T-maze testing. Remaining food was measured following testing and caloric intake calculated. All diets were coded and all researchers were blinded to dietary treatment.

### 2.3. Body Composition

Body weight was measured weekly prior to the morning feeding. Body composition was measured by dual X-ray absorptiometry (DXA; Hologic Inc.) as previously described on week −1 and week 16 [10]. Cats with a BW of less than 4.0 kg were not assessed, due to minimum weight allowances of the software, and resulted in exclusion of 7 cats among the two treatment groups.

### 2.4. Indirect Calorimetry

To assess the effects of LC treatment on energy expenditure (EE) and respiratory quotient (RQ), indirect calorimetry was utilized and conducted as previously described at baseline and at weeks 4, 6, 10, and 14 on cats that had been acclimated to temporary restriction and the calorimetry chambers [7, 15]. Air from each chamber and background air samples were measured for 5 minutes every half hour for a total of 22 h. Background air was used to correct for CO_2_ and O_2_ of incoming room air. Oxygen (VO_2_) consumed and carbon dioxide (VCO_2_) produced were measured. Concentrations of O_2_ and CO_2_ were measured with infrared and O_2_ and CO_2_ analyzers (Qubit Systems^®^, Kingston, Ontario, Canada). The calorimeter is an open circuit, ventilated calorimeter with the room air being drawn through at a rate of 5–10 L/min depending on the body weight of the cat. The rate of airflow was measured with the use of a mass flow meter to enable total volume to be calculated. Calibration of the analyzers and mass flow meters were performed prior to each oxidation study and every 6 hours or when a drift of >5% was observed. Calibration was performed using standard gas mixtures against known calibration standards.

To calculate RQ, EE, fat, and carbohydrate oxidation the following calculations were used:

 RQ = liters of CO_2_ produced/liters of O_2_ consumed.
 Resting EE (kcal) = 3.82 × liters of O_2_ consumed + 1.15 × liters of CO_2_ produced [16].

### 2.5. Behavioral Assessments

Voluntary physical activity was measured using the validated Actical Activity Monitors (Mini Mitter, Bend, OR, USA) over 5 consecutive 24 h periods (Monday to Friday) during weeks −1 (baseline) and 12 when no other collections occurred.

To assess play motivation, an obstruction test was used to measure willingness to work to gain access to a valued toy with the swing door made progressively more difficult to open through the addition of weights (50 g) that were placed into a trough at the bottom of the door (max 600 g). Cats were assessed at baseline and at weeks 1 and 15 of treatment at approximately 5 h after feeding.

A T-maze (stem: 7′ L × 1.5′ W × 1.5′ H; arm: 3.25′ L × 1.5′ W × 1.5′ H) was used to measure cognitive function 6 h after feeding at baseline and at weeks 2, 8, and 16 of the study. A spatial cue (a circular shape and an X shape) was randomly assigned as a positive (rewarded) and negative (nonrewarded) cue for each cat and balanced for diet. Ten trials per day were used to measure number of correct arm entries. Both arms were baited with 1 g of food to ensure that olfactory cues did not influence performance; however, food was only accessible to cats if they entered the correct arm containing that cat's positive (rewarded) cue.

### 2.6. Statistical Analyses

All data were analyzed using SAS version 9.2 (SAS Institute, Cary, NC) and results expressed as means and standard errors of the means. Sample size was estimated using energy expenditure from our previous study [7] at a difference of 3%, a power of 80%, and *α* of 0.05. We used these data because there is no data in which cats are in positive energy balance and energy metabolism has been measured and this calculation may have been inappropriate. All data were checked for normality. Results were considered at *P* < 0.05. The calorimetry (area under the curve, AUC), food intake, and median latency to enter the T-maze junction data were analyzed using a repeated analyses of covariance model with baseline outcomes used as a covariate to investigate differences between dietary treatments. The calorimetry analysis was done separately for the fasted period and the entire time the cat was in the oxidation chamber (AUC from 0 to 21 hours after feeding). Maximum door weight (play motivation) and body weight were analyzed using the same model as the calorimetry data with the addition of baseline body weight as a covariate. An ANCOVA model was used to analyze DXA (global fat, global lean, and fat-to-lean ratio) and activity (intensity and duration) data, and baseline body composition was included as a covariate. A day/night indicator variable was added to the activity models. The proportion of correct T-maze arm entries was analyzed using a repeated measures logistic regression model.

## 3. Results

### 3.1. Feed Intake

**Figure 1:**
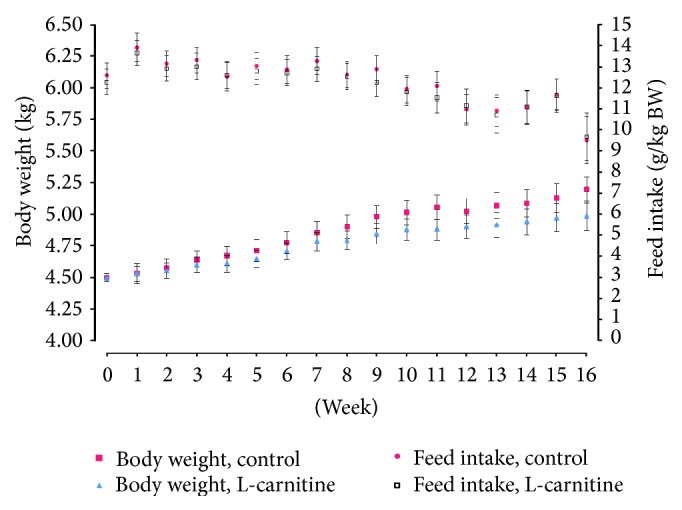
Mean (±SEM) food intake (g/kg/d) and BW (kg) in cats fed LC121 or LC188 receiving 120% of their maintenance energy requirement on a per kg basis during 16-week feeding.

There was no effect of dietary treatment on total food intake on a g/kg body weight basis over the course of the study (*P* = 0.835). Over the 20-week feeding period, food intake significantly decreased, as a function of BW, in both groups (Figure 1; *P* < 0.0001) despite food allowance being altered based on body weight every week.

### 3.2. Body Composition and Body Weight

Cats that were fed both diet treatments gained weight throughout the 16-week feeding period (*P* < 0.0001), but treatment groups did not differ from each other at any point throughout the study (*P* > 0.05; Figure 1) and there was no time ∗ treatment effect (*P* > 0.05). There were no differences in global or absolute adipose or lean body mass between treatment groups at baseline (*P* > 0.05; Table 2). Global body fat (*P* = 0.047) and global fat : lean ratio (*P* = 0.038) significantly increased through the duration of the study for both treatment groups (*P* < 0.05), but lean body mass did not change through the duration of the study for either treatment group (*P* > 0.05). Furthermore, there was a time ∗ treatment effect (*P* < 0.05) for adipose and global fat : lean ratio (*P* > 0.05). Gains in adipose and global fat : lean ratio were significantly less for cats consuming LC188 versus cats consuming LC121 (*P* < 0.05).

### 3.3. Fasted Energy Metabolism

Fasted RQ was significantly lower in cats fed LC188 at baseline (*P* = 0.05) and used as a covariate to generate adjusted LS means for RQ in the subsequent analyses for weeks 4, 6, 10, and 14. Respiratory quotient did not differ between groups in the fasted state (*P* = 0.232; Table 3). Energy expenditure (kcal/(kg∗d)) in the fasted state did not differ between groups at baseline and at week 4, 6, 10, or 14 (Table 3; *P* > 0.05). A significant effect by week was detected (*P* < 0.05), but there was no treatment ∗ week interaction.

### 3.4. Fed Energy Metabolism

Postmeal mean AUC for RQ did not differ between treatment groups at baseline or at any point during the study (Table 3; *P* > 0.05). When data were analyzed over the postprandial (0–5.25 h), fed (5.25–10.50 h), return to fasted (10.50–15.75 h), and fasted (15.75–21 h) states, there were no effects of diet on RQ (*P* > 0.05,* data not shown*). There was no effect of diet on postmeal mean AUC for EE (kcal/(kg∗d); Table 3; *P* > 0.05). Energy expenditure analyzed over the postprandial (0–5.25 h), fed (5.25–10.50 h), return to fasted (10.50–15.75 h), and fasted (15.75–21 h) states did not differ between groups (*P* > 0.05,* data not shown*) at baseline or at any point throughout the study. A significant effect by week was detected (*P* < 0.05), but there was no treatment ∗ week interaction.

### 3.5. Behavioral Assessments

Voluntary physical activity did not differ between groups for total movement (*P* = 0.527) and time spent moving (*P* = 0.610) or when the data were split between daytime and night activity (*P* > 0.05, Table 4). Performance during T-maze testing did not differ between dietary treatments (*P* = 0.219, Table 5). Mean latency to make a selection for the T-maze was not different between cats fed LC188 and LC121 (*P* = 0.711). There was no effect of diet on play motivation or mean weight and the cats were willing to push open to gain access to a toy (LC121 = 292.45 g ± 62.82 and LC188 = 339.58 ± 74.27; *P* = 0.626).

## 4. Discussion

Cats in positive energy balance fed diets containing 188 ppm LC have lower body fat deposition than cats fed diets containing 121 ppm LC in a 16-week feeding study. Despite these significant changes in body composition, there were no differences in LC treatments for measures of energy metabolism, or physical activity; therefore, the mechanism of action for the reduced adipose gain with the 188 ppm LC fed cats remains unclear. Furthermore, there was no effect of diet on any behavioral outcomes including play motivation and cognitive performance, although the study was not powered against outcomes of physical activity or behavior, which are more variable than more physiological outcomes. Overall, feeding LC, particularly higher doses, to cats in positive energy balance may be beneficial to help mitigate weight and more importantly adipose gain.

The effect of decreased adipose gain in cats fed LC188 is not surprising. The presence of LC in 3T3-L1 adipocyte culture resulted in increased hormone-sensitive lipase, carnitine palmitoyltransferase I-a, and acyl-coenzyme A oxidase, suggesting that LC may act to increase lipid oxidation [17]. Furthermore, the expressions of peroxisome proliferator-activated receptor-*γ* and adipose-specific fatty acid binding protein were downregulated by LC in 3T3-L1 adipocytes, suggesting a decrease in adipogenesis [17], which supports the findings in the current study. Similarly, a cocktail of red grape extract, soy isoflavone, and LC was found to inhibit body weight and adipose gain in C57BL/6J mice consuming a high fat diet [18], although the use of a cocktail including other compounds is difficult to compare to the current study. Another cocktail of* Garcinia cambogia*, soy peptide, and LC reduced visceral fat accumulation in Sprague Dawley rats fed high fat diets [19] but again is not solely the action of LC. Similarly, a cocktail of an Egyptian herbal formula and LC resulted in reduced body weight gained and a better metabolic profile compared to rats that did not receive the supplement [20]. A longer treatment period and/or more animals may be necessary to elucidate the positive effects on biomarkers and warrants further investigation. This same experimental paradigm approach combined with ad libitum feeding may also demonstrate significant effects on total body weight gain and warrants further investigation. Indeed, providing a nutritional technology that could mitigate weight gain, especially in households with multiple cats, would significantly help owners manage individual cat body weight.

Although we observed a difference between diets on adipose tissue deposition, there were no effects of diet on EE or metabolic fuel selection as underlying mechanisms for the observed compositional changes. Previous data collected in cats fed supplemental LC have been completed during maintenance or restricted (for weight loss) feeding paradigms in which LC supplementation contributes to an increase in EE and fatty acid oxidation [7, 9]. Feeding above energy requirements in the current study did not produce similar results as there were no differences in EE and RQ between cats fed the diet with 121 ppm LC and cats fed the diet with 188 ppm LC. As cats fed both treatments gained weight, fasting EE (kcal/kg BW/d) decreased, but overall there was no difference between dietary treatments. There was a significant response over time in fasting RQ, but there was no difference between diets. Fasting RQ and postprandial EE and RQ appeared to follow a less clear pattern over time. The lack of metabolic response within a feeding paradigm above energy requirements may be due to (1) the overriding effect of total caloric intake on LC effectiveness and (2) the theory that the population of animals that would mostly benefit from LC treatment are those with LC deficiency, namely, cats undergoing weight loss, which are at risk for the development of hepatic lipidosis [5, 9, 21].

Overall, the dietary concentration of LC within the diet had no effect on voluntary physical activity, motivation to play, and performance during a T-maze test. Several studies have reported that treatment with LC in populations with LC insufficiency, primarily due to disease or fatigue syndrome, can lead to reductions in subjective feelings of low energy [22–24]. Further, supplementation with acetyl-L-carnitine and LC has been shown to increase voluntary physical activity levels when fed to aged rats [14]. However, the effect of LC on ambulatory behavior is not always apparent [24], which is consistent with our findings, as we did not observe an effect of LC on physical activity in healthy, adult cats. Previously, LC supplementation at 100 mg/kg contributed to an increased motivation to play in overweight cats (BCS > 3.5) [7], but a similar response was not observed within the current study. The lack of effect of LC on play motivation may have been due to the absence of effect on energy metabolism (particularly EE) which has historically been linked to increases in play motivation in cats [7]. Similar to previous studies in humans and animals, the effects of LC may be more apparent in populations at risk for LC insufficiency or those exhibiting declines or impairments in cognitive function, activity, and play. Indeed, the cats used in the present study were healthy and young and received a lot of cat-cat and human socialization and may not be the suitable cohort to observe effects on play motivation or physical activity.

In conclusion, these results suggest that diets containing 188 ppm LC and fed above maintenance energy requirements will result in less fat gain than diets with 121 ppm LC and may be beneficial for the health and well-being of cats that may be overfed. However, LC may be required at different levels depending on total caloric intake and level of adiposity, and a dose-dependent comparison in multiple experimental paradigms is necessary to elucidate the mechanism of action of LC on adipose gain and how the individual physiological state (e.g., level of adiposity) of cats affects the expected outcome.

## Figures and Tables

**Table 1:** Guaranteed analysis on an “as fed” basis of final products fed to cats. All analyses represent an average of triplicate analyses.

Nutrient	LC121 (121 ppm LC)	LC188 (188 ppm LC)
Fat (%)	16.2	15.8
Ash (%)	6.2	6.0
Crude fiber (%)	1.75	2.07
Moisture (%)	6.11	6.23
Protein (%)	33.5	32.3
L-carnitine (ppm)	121	188
Calculated energy density (kcal/kg)^*∗*^	3752	3728

^*∗*^Value calculated using the modified Atwater calculation and not accounting for total dietary fiber.

**Table 2:** Mean (±SEM) absolute or global fat (g), lean (g), and fat/lean ratio in adult cats fed LC121 or LC188 supplemented diets modified ad libitum receiving 120% of their estimated daily energy requirement.

		LC121 (*N* = 6)	LC188 (*N* = 7)	Pairwise *P* value
Global fat (g)	Baseline	794.1 ± 255.4	1044.6 ± 236.4	0.49
	Week 16	1460.4 ± 112.1	1106.9 ± 110.6	0.047

Global lean (g)	Baseline	4112.5 ± 218.5	4029.9 ± 202.3	0.79
	Week 16	4242.9 ± 74.7	4285.4 ± 65.8	0.67

Global fat/lean ratio	Baseline	0.189 ± 0.056	0.257 ± 0.052	0.40
	Week 16	0.353 ± 0.028	0.259 ± 0.027	0.038

**Table 3:** Fasting and fed adjusted AUC for RQ and EE in adult cats fed LC121 or LC188 supplemented diets above their maintenance energy requirement at baseline and during the 16-week LC feeding.

	Week	LC121 ± SE (*N* = 10)	LC188 ± SE (*N* = 10)	Pairwise *P* value
Fasting RQ^*∗*^	Baseline	0.756 ± 0.006	0.740 ± 0.006	0.05
	4	0.785 ± 0.013	0.776 ± 0.012	0.61
	6	0.811 ± 0.014	0.784 ± 0.013	0.16
	10	0.839 ± 0.013	0.821 ± 0.012	0.33
	14	0.783 ± 0.015	0.763 ± 0.010	0.26

Fasting EE (kcal/(kg*∗*d))^*∗*^	Baseline	45.27 ± 3.78	47.81 ± 3.78	0.64
	4	42.91 ± 4.09	43.16 ± 2.02	0.96
	6	41.15 ± 4.07	40.53 ± 2.42	0.89
	10	37.29 ± 3.86	37.06 ± 2.53	0.96
	14	40.52 ± 4.48	39.05 ± 1.87	0.78

Postprandial RQ^*∗*^	Baseline	0.81 ± 0.007	0.80 ± 0.007	0.20
	4	0.82 ± 0.008	0.81 ± 0.007	0.22
	6	0.86 ± 0.008	0.85 ± 0.009	0.33
	10	0.86 ± 0.008	0.85 ± 0.009	0.33
	14	0.81 ± 0.008	0.80 ± 0.01	0.38

Postprandial EE (kcal/(kg*∗*d))^*∗*^	Baseline	42.97 ± 3.25	44.69 ± 3.25	0.72
	4	41.84 ± 1.81	43.10 ± 1.69	0.58
	6	35.24 ± 1.72	34.73 ± 1.14	0.81
	10	33.52 ± 1.77	33.21 ± 1.32	0.89
	14	41.37 ± 2.58	42.46 ± 1.13	0.70

*∗* denotes a significant (*P* < 0.05) time effect.

**Table 4:** Adjusted means (±SEM) for physical activity in adult cats fed LC121 and LC188 supplemented diets above maintenance energy requirements.

Parameter	Period^*∗*^	LC121 (*N* = 9)	LC188 (*N* = 10)	Pairwise comparison *P* value
Physical activity intensity (activity counts per hour)	Total	1244 ± 88	1328 ± 103	0.52
	Light	1594 ± 83	1599 ± 76	0.96
	Dark	985 ± 134	1158 ± 155	0.39

Physical activity duration (proportion of activity time within a day)	Total	0.097 ± 0.007	0.100 ± 0.004	0.61
	Light	0.117 ± 0.007	0.117 ± 0.006	0.98
	Dark	0.081 ± 0.008	0.088 ± 0.006	0.46

^*∗*^Light and dark were defined in accordance with the sunrise and sunset time.

**Table 5:** T-maze testing and proportion of correct arm entries (baseline adjusted mean (SE)) of cats fed LC121 or LC188 diets.

	LC121 (*N* = 10)	LC188 (*N* = 10)	*P* value
Week 2	0.64 (0.05)	0.73 (0.05)	0.265
Week 8	0.73 (0.07)	0.76 (0.05)	0.737
Week 16	0.76 (0.05)	0.80 (0.04)	0.533

Overall	0.72 (0.03)	0.76 (0.02)	0.219

## Abbreviations

BCS: Body condition score
BW: Body weight
EE: Energy expenditure
RQ: Respiratory quotient
ME: Metabolizable energy.
